# Identification of *STRA6* and *SKI* sequence variants in patients with anophthalmia/microphthalmia

**Published:** 2008-12-26

**Authors:** Tristan White, Tianyi Lu, Ravikanth Metlapally, James Katowitz, Femida Kherani, Tian-Yuan Wang, Khanh-Nhat Tran-Viet, Terri L. Young

**Affiliations:** 1 The Center for Human Genetics, Duke University Medical Center, Durham, NC; 2 Department of Ophthalmology, Duke University Medical Center, Durham, NC; 3 Children’s Hospital of Philadelphia, Division of Ophthalmology, Philadelphia, PA

## Abstract

**Purpose:**

Anophthalmia and microphthalmia (A/M) are rare congenital ocular malformations presenting with the absence of eye components or small eyes with or without structural abnormalities. A/M can be isolated or syndromic. The stimulated by retinoic acid gene 6 (*STRA6*) and Sloan-Kettering viral oncogene homolog (*SKI*) genes are involved in vitamin A metabolism, and are implicated with A/M developmental abnormalities in human and animal studies. Vitamin A metabolism is vital to normal eye development and growth. This study explores the association of these genes in a cohort of subjects with A/M.

**Methods:**

*STRA6* and *SKI* were screened for sequence variants by direct sequencing of genomic DNA samples from 18 affected subjects with A/M. The DNA samples of 4 external, unrelated controls were initially screened. Eighty-nine additional unrelated controls were screened to confirm that any sequence variants found in the affected subject DNA samples were related to the phenotype. Coding regions, intron-exon boundaries, and untranslated regions were sequenced by standard techniques. Derived DNA sequences were compared to known reference sequences from public genomic databases.

**Results:**

For *STRA6*, a novel coding non-synonymous sequence variant was found in one subject, resulting in an amino acid change from glycine to glutamic acid in residue 217. One novel nonsense sequence variant found in the same subject changed the *STRA6* amino acid residue 592 from cytosine to thymine resulting in a premature stop codon. For *SKI*, a known coding non-synonymous sequence variant (rs28384811) was found in 3 subject DNA samples and 11/89 control DNA samples. Four novel coding-synonymous sequence variants were observed in *SKI*.

**Conclusions:**

The *STRA6* sequence variants reported in this study could play a role in the pathogenesis of A/M by structural changes to the *STRA6* protein. We can attribute 4% A/M incidence in this cohort to these sequence variants. Although no *SKI* sequence variants were found in this cohort, *SKI* should not be ruled out as a candidate gene for A/M due to the small cohort size.

## Introduction

Anophthalmia and microphthalmia (A/M) are rare congenital ocular malformations presenting with the absence of eye components (anophthalmia) or small eyes with or without structural abnormalities (microphthalmia). A/M has a prevalence of approximately 30 per 100,000 population [1-3], or 70 incidents per 480,000 live births in the United States [4]. A/M may be isolated or syndromic with varied patterns of inheritance. Many genetic loci are associated with either isolated or syndromic A/M. To date, there are 3 genetic loci identified for isolated A/M; MCOP1 (14q32; OMIM 251600), MCOP2 (14q24.3; OMIM 610093), MCOP3 (18q21.3; OMIM 601881). In addition, there are 10 loci identified for syndromic A/M; MCOPS1 (Xq27–28; OMIM 309800), MCOPS2 (Xp11.4; OMIM 300166), MCOPS3 (3q26.3-q27; OMIM 206900), MCOPS4 (Xq27–28; OMIM 301590), MCOPS5 (14q21–22; OMIM 610125), MCOPS6 (14q22–23; OMIM 607932), MCOPS7 (Xp22; OMIM 309801) MCOPS8 (6q21; OMIM 601349), MCOPS9 (15q24.1; OMIM 601186), and MCOPS10 (OMIM 601186). There are other syndromes or genes that have been associated with A/M; CHARGE Syndrome (8q12.1, 7q21.1; OMIM 214800), Frasier Syndrome (13q13.3, 4q21; OMIM 219000), and the paired box gene 6 (*PAX6*; 11p13; OMIM 607108). The large number of loci associated with A/M reflects the heterogeneity of this ocular malformation.

Current research in determining the genetic etiology of A/M has primarily focused on known gene mutations in SRY - (sex determining region Y)-box 2 (*SOX2*; OMIM 184429), paired box gene 6 (*PAX6*; OMIM 607108), orthodenticle homeobox 2 (*OTX2*; OMIM 600037), *C. elegans* ceh-10 homeo domain containing homolog (*CHX10*; OMIM 142993), and retina and anterior neural fold homeobox (*RAX*; OMIM 601881). *SOX2*, *OTX2*, and *PAX6* are all necessary for ocular development [5]. *OTX2*, *PAX6*, and *SOX2* are also involved in vitamin A metabolism either directly or through complex gene-gene interactions [6-8]. The present cohort studied was screened for *SOX2* and *CHX10* sequence variants in a previous study [9], and no pathogenic *SOX2* or *CHX10* mutations were found.

**Figure 1:**
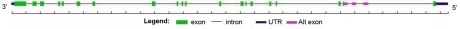
The *STRA6* gene structure showing the intron-exon layout. The *STRA6* gene comprises 20 exons, with several alternate first exons, coding for a protein 667 amino acids in length. Generated from the Gene Structure Display Server.

Although various mutations and deletions of the genes listed above are implicated in many cases of A/M, these genes do not account for all cases of A/M. Recently, homozygous mutations in the stimulated by retinoic acid gene 6 (MCOPS9; *STRA6*) [10,11] were identified in families with Matthew-Wood syndrome, an A/M phenotype associated with pulmonary hypoplasia and cardiovascular malformations [12]. *STRA6* maps to chromosome 15q.24.1 and is a 20 xon gene encoding a protein of 667 amino acids (Figure 1). *STRA6* encodes for a trans-membrane receptor for the retinol binding protein (RBP) which enhances cellular uptake of vitamin A or retinol [13]. RBP is necessary for the transport of retinol to specific sites in the eye, such as the retinal pigment epithelium layer and retinal vasculature [13]. Following the uptake into the cell, retinol is oxidized to retinal which is in turn oxidized to retinoic acid (RA) [14]. Vitamin A and its derivatives are important substrates for proper ocular development and other cellular functions [14-16].

**Figure 2:**
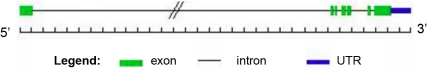
*SKI* gene structure showing the intron-exon layout of the 7-exon gene. The *SKI* gene comprises 7 exons which code for a 728 amino acid long protein. Generated from the Gene Structure Display Server.

In a recent report, C57BL/6J mice lacking the Sloan-Kettering viral oncogene homolog (*Ski*) gene had a complex ocular phenotype that included microphthalmia, hyperplastic hyaloid vasculature, and severe abnormalities of the iris ranging from aniridia to iris coloboma [17,18]. *SKI* maps to chromosome 1p36.3 and is a 7 xon gene encoding a protein of 728 amino acids (Figure 2). The *SKI* gene primary domain interacts with the small mothers against decapentaplegic proteins (*SMAD*) to repress the transcription of transforming growth factor-beta (*TGF-β*) genes [19]. The *TGF-β g*ene family is a major regulator of cellular functions such as cell proliferation, apoptosis, specification, development fate, and ocular growth [20,21]. *SKI* is also an activator of *MITF*, a gene implicated in microphthalmia [19]. Nuclear *SKI* binds to the active retinoic acid receptor (RAR) complex and recruits co-repressors inhibiting transcription mediated by the active RAR complex [22]. *SKI* inhibits RAR signaling through different pathways including the brain all-trans-retinoic acid signaling, *TGF-β*, and bone morphogenetic protein pathways [22,23].

*STRA6* and *SKI* are candidate genes associated with the A/M phenotype and are involved in vitamin A metabolism and RA signaling. We hypothesized that sequence variants in either *STRA6* or *SKI* may give rise to the A/M phenotype. In this study, a cohort of 18 patients with A/M was screened for sequence variants in *STRA6* and *SKI*.

## Methods

### Subjects

Informed consent was obtained from all participants. The study followed the principles of the Declaration of Helsinki, and was approved by the Institutional Review Board at the Duke University Medical Center. Eighteen A/M probands and 4 external control subjects (all unrelated) were initially screened to identify known and novel sequence variants. Subject demographics are listed in Table 1. Genomic DNA was extracted via venous blood using AutoPure LS^®^ DNA Extractor and PUREGENE™ reagents (Gentra Systems Inc., Minneapolis, MN). Novel coding non-synonymous and nonsense sequence variants identified in a proband were further tested in 89 external control DNA samples.

**Table 1:** Subject demographics and clinical descriptions.

**Subject ID**	**Age at ascertainment**	**Gender**	**Race**	**Ocular phenotype**	**Systemic findings**	**Family history**
1	3yrs 2 months	M	Caucasian	Bilateral microphthalmia	Cleft lip and palate Developmental delay	Adopted
2	3yrs	M	Caucasian	Anophthalmia/Microphthalmia	None	Brother with A/M
3	7 months	F	Caucasian	Unilateral microphthalmia	None	None
4	11 days	F	Caucasian	Bilateral microphthalmia	46, XX, der (4) t (2,4) (q31.1;q33) Choanal atresia, congenital heart disease, lung hypoplasia, and developmental delay.	None
5	28 years 1 months	M	Caucasian	Unilateral microphthalmia	None	None
6	7 months	M	African American	Bilateral microphthalmia	None	None
7	5 years 6 months	F	Caucasian	Bilateral microphthalmia	None	None
8	1 year 4 months	M	Caucasian	Microphthalmia OS	None	None
9	6 years 5 months	M	Caucasian	Microphthalmia OD	None	None
10	1 year 7 months	F	Caucasian	Microphthalmia OS	None	None
11	6 years 9 months	F	Caucasian	Bilateral anophthalmia	Duplicated kidney collecting system	None
12	3 years 5 months	M	Caucasian	Bilateral microphthalmia	Cleft lip and palate	Adopted
13	6 years 3 months	M	Caucasian	Bilateral microphthalmia	Scimitar syndrome (anomalous pulmonary venous system)	None
14	15 years 1 months	F	Asian	Bilateral anophthalmia	Ventricular-septal defect. Fused tooth.	None
15	2 years 6 months	F	Caucasian	Bilateral microphthalmia	Hypotonia, tremors, lax ligaments	Mother with polar cataracts
16	4 months	F	Hispanic	Microphthalmia OD Anophthalmia OS	None	None
17	2 years	M	Caucasian	Bilateral anophthalmia	None	None
18	4 years 2 months	M	Caucasian	Bilateral anophthalmia	None	None

All cases were post-natal, and there was no known parental consanguinity. OD=Right eye, OS=Left eye.

### Polymerase chain reaction (PCR) and DNA sequencing

Seventeen and twelve primer sets were designed to amplify the 19 exons of *STRA6* and the 7 exons of *SKI,* respectively. Each amplicon extended 50–100 base pairs beyond the intron-exon boundary and the 5′ and 3′ untranslated regions (UTRs) of the genes. Amplification of the 5′ genomic sequence of *SKI* exon 1 using multiple primer sets was unsuccessful due to high GC content. PCRs run using either a normal reaction or a 1M Betaine (Sigma-Aldrich, Saint Louis, MO) reaction were performed on genomic DNA from the 18 A/M patients and 4 external controls using Platinum® Taq DNA polymerase (Invitrogen Corporation, Carlsbad, CA). Amplicons were visualized after electrophoresis on a 2% agarose gel and purified using Quickstep™ 2 SOPE™ Resin (Edge BioSystems, Gaithersburg, MD). Sequencing reactions were performed using BigDye™ Terminator v1.3 and run on an ABI3730 Sequencer (Applied Biosystems, Inc., Foster City, CA). Sequences were trimmed for quality and aligned with the corresponding reference sequences (i.e., *STRA6* [NM_022369] and *SKI* [NM_003036]) using the Sequencher™ program (Gene Codes, Ann Arbor, MI). DNA sequences were analyzed and compared to determine sequence variants.

## Results

**Figure 3:**
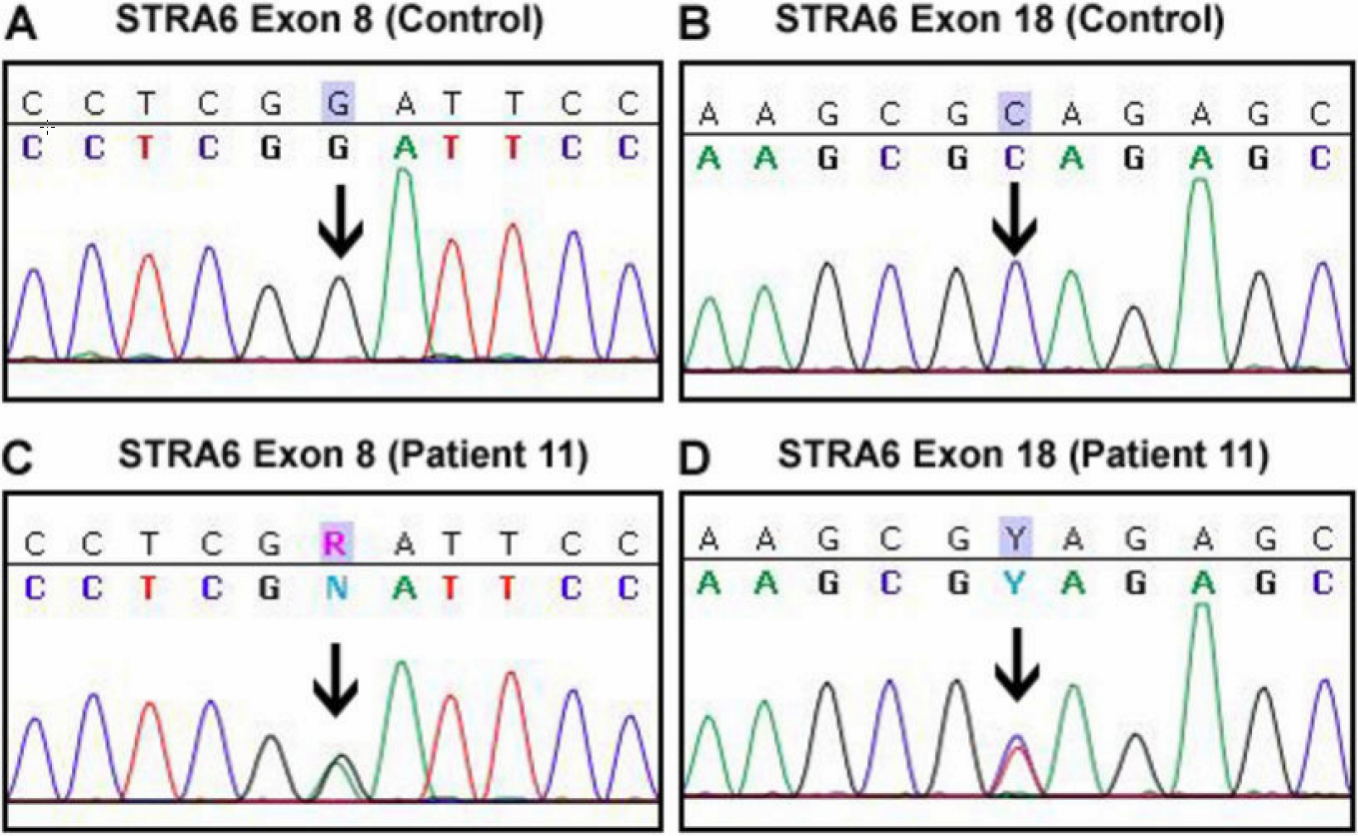
Chromatograms showing *STRA6* sequence variants in subject 11. **A** and **C** display the novel missense sequence variant in subject 11 in exon 8. This base pair sequence change of guanine to adenine causes an amino acid change from glycine to glutamic acid at residue 217. **B** and **D** display the novel nonsense sequence variant in subject 11 in exon 18. This sequence variant changes the glutamine amino acid residue at position 592 and produces a premature stop codon.

Twenty-two sequence variants (1 nonsense, 2 missense, 1 silent, 6 UTR, 10 intronic, 1 intronic deletion, and 1 intronic insertion) were identified in *STRA6*. Among them, 7 were novel, including 1 missense and 1 nonsense sequence variant. The novel missense sequence variant (Figure 3) in exon 8 of *STRA6* was noted in subject 11. This guanine to adenine sequence variant changes amino acid residue 217 from glycine to glutamic acid (Table 2). A novel nonsense sequence variant (Figure 3) in exon 18 was also noted for the same subject (Table 2). The nonsense sequence variant changes amino acid residue 592 from cytosine to thymine resulting in a premature stop codon. Neither sequence variant was detected in 89 external control DNA samples. No other sequence variants were associated with the disease status. All non-coding *STRA6* sequence variants observed are reported in Appendix 1.

**Table 2:** Sequence variants in *STRA6* determined by direct sequencing.

**Patient ID**	**Exon 5**	**Exon 8**	**Exon 17**	**Exon 18**
	rs11857410	**Novel**	rs736118	**Novel**
	**Coding synonymous**	**Coding non-synonymous**	**Coding non-synonymous**	**Nonsense-stop codon**
1				
2				
3	c.[331C>T ] +[331C>T ], p.L111L		c.[1581G>A]+[=], p.[527M>I]+[=]	
4				
5	c.[331C>T ] +[=], p.L111L			
6				
7			c.[1581G>A]+[=], p.[527M>I]+[=]	
8	c.[331C>T ] +[331C>T ], p.L111L			
9				
10	c.[331C>T ] +[=], p.L111L			
11		*c.[650G>A]+[=], p.[217G>E]+[=]		*c.[1774C>T]+[=], p.[592Q>X]+[=]
12			c.[1581G>A]+[=], p.[527M>I]+[=]	
13			c.[1581G>A]+[=], p.[527M>I]+[=]	
14			c.[1581G>A]+[=], p.[527M>I]+[=]	
15				
16				
17	c.[331C>T ] +[=], p.L111L			
18				
Control 1	c.[331C>T ] +[=], p.L111L			
Control 2	c.[331C>T ] +[=], p.L111L			
Control 3				
Control 4	c.[331C>T ] +[=], p.L111L		c.[1581G>A]+[=], p.[527M>I]+[=]	

The 2 *STRA6* sequence variants following affection status are marked with asterisks, the novel exon 8 coding non-synonymous and the novel exon 18 nonsense sequence variants. These sequence variants were not detected in 89 control samples.

For *SKI*, 20 sequence variants were identified; 1 missense, 5 silent, 9 intronic, 4 UTR, and 1 intronic deletion. Thirteen of the 20 sequence variants found in *SKI* were novel. A known coding non-synonymous sequence variant, rs28384811, was found in 3 affected subject DNA samples (Table 3). This sequence variant changes amino acid residue 62 from alanine to glycine. However, this sequence variant was also found in 11 out of 89 control DNA samples screened. No other *SKI* sequence variants were found to be significant. All non-coding *SKI* sequence variants observed are reported in Appendix 2.

**Table 3:** Sequence variants in *SKI* determined by direct sequencing.

**Patient ID**	**Exon 1**	**Exon 1**	**Exon 4**	**Exon 4**	**Exon 5**	**Exon 6**
	rs28384811	**Novel**	**Novel**	**Novel**	**Novel**	**Novel**
	**Coding non-synonymous**	**Coding synonymous**	**Coding synonymous**	**Coding synonymous**	**Coding synonymous**	**Coding synonymous**
1						
2						
3						
4						
5					c.[1527C>T] +[=], p.A509A	
6						
7						
8						
9	*c.[185C>G] +[=], p.[62A>G]+[=]	c.[98C>G] +[=], p.G33G				
10	*c.[185C>G] +[185C>G], p.[62A>G] +[62A>G]					
11	*c.[185C>G] +[185C>G], p.[62A>G] +[62A>G]	c.[98C>G] +[98C>G], p.G33G				
12						
13						
14			c.[1440G>A] +[=], p.S480S			
15				c.[1446G>A] +[=], p.A482A		
16						
17						
18						
Control 1						c.[1974C>T] +[=], p.R658R
Control 2						
Control 3						
Control 4						

The coding non-synonymous sequence variant (rs28384811) marked with an asterisk was also detected in 11 out of 89 control DNA samples.

## Discussion

The developmental eye disorders anophthalmia and microphthalmia may involve several genes. Identifying causative genes will not only help improve understanding of the disease process, it will also provide insight into ocular growth and development of the eye in general. In this study, we screened the candidate genes *SKI* and *STRA6* in an A/M cohort. Neither gene has been screened in a relatively non-syndromic cohort of A/M patients to date. We report 1 nonsense and 1 missense sequence variant in 1 affected subject (#11) in *STRA6*. These sequence variants were not seen in 89 external control samples. Subject 11 is a Caucasian female who was 6 years and 9 months old at the time of ascertainment. There was no reported parental consanguinity. The subject had bilateral microphthalmia and a duplicated kidney collecting system as the only structural abnormalities noted. Subject 11 did not have the cardinal features of Mathew-Wood Syndrome (MCOPS9), such as cardiopathy, lung hypoplasia, and diaphragmatic hernia. We believe that the A/M of subject 11 is non-syndromic, and that the *STRA6* sequence variants reported may be associated with this phenotype.

**Figure 4:**
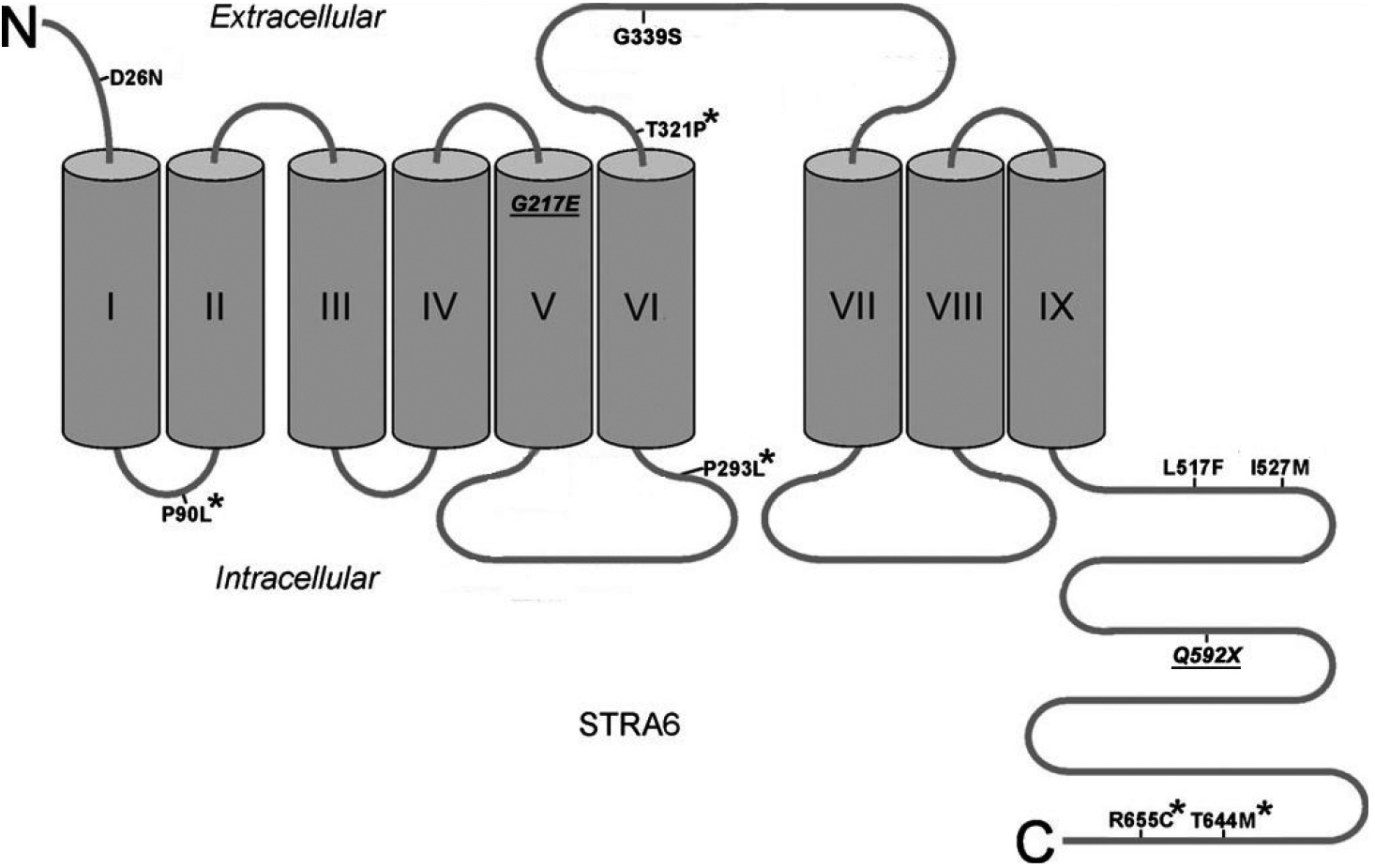
Diagram of the STRA6 protein structure showing transmembrane regions, and intracellular and extracellular domains. (Figure modified from Kawaguchi et al. [24], provided with the author’s permission). Underlined sequence variants were found in this study. The missense sequence variant at amino acid 217 changes a hydrophobic glycine residue to a hydrophilic glutamic acid residue located in a hydrophobic transmembrane region. The nonsense sequence variant at amino acid 592 causes a premature stop codon on the COOH-terminus end of the STRA6 protein. Previously discovered sequence variants marked with asterisks are involved in human disease. Other sequence variants are missense mutations annotated in the GenBank database, and are not currently known to be associated with human disease.

The novel nonsense sequence variant in *STRA6* changes the glutamine residue at position 592 to produce a premature stop codon. This stop codon cleaves the predicted COOH-terminal domain of the protein. This highly conserved long COOH-terminal domain is located intracellularly (Figure 4), and is speculatively involved in vitamin A uptake [24]. Thus, the cellular uptake of vitamin A may be affected by a truncated STRA6 protein in the phenotype involved. Normal STRA6 function is critical for vitamin A uptake into cells, especially during embryonic development [25]. Little or no STRA6 function leads to severe phenotypes including ocular defects [10,11]. There are 2 known missense mutations in the COOH-terminal domain associated with human anophthalmia (Figure 4) [10]. These 2 known mutations disrupt the normal function of the C-terminal domain of the STRA6 protein, so it follows that premature termination of the COOH-terminal domain would also have a profound effect on the function of STRA6.

The missense sequence variant observed in subject 11 alters amino acid 217 from a hydrophobic glycine residue to a hydrophilic glutamic acid residue. This polarity change occurs in a highly conserved and hydrophobic transmembrane region, the 5th predicted in the STRA6 protein (Figure 4) [24]. This is likely to have effects on protein folding. Computational analysis by the PolyPhen web tool predicted the amino acid change from glycine to glutamic acid to be possibly damaging, citing an improper substitution in a transmembrane region. A similar change was observed by Kawaguchi et al. [25] in the STRA6 transmembrane region 6 (valine to glutamic acid) resulting in a severe reduction of vitamin A uptake. We speculate that such disrupted vitamin A metabolism, especially during development, is involved in the A/M phenotype. Viable cell lines were not available for subject 11 to confirm the nonsense and missense sequence variants at the mRNA level. Parental DNA for subject 11 was also unavailable to determine the origin of these two potentially pathogenic variants.

*SKI* is also involved in the RA pathway, however unlike *STRA6* no *SKI* sequence variants segregated with affection status. The known missense sequence variant (rs38284811) found in 3 affected individuals changes amino acid residue 62 from alanine to glycine. This sequence variant may not be responsible for the A/M phenotype, as it was also found in 11 out of 89 external controls screened. *SKI* should not be ruled out as a candidate gene for A/M, as the cases studied may not cover the entire A/M phenotype spectrum.

The limitation of this study is the small cohort size screened. The small cohort size and diverse nature of the disease phenotypes studied in this cohort make it difficult to extrapolate the incidence rate of *STRA6* mutations in the general A/M population. However, it is difficult to collect large, homogenous sample sets of isolated A/M patients. In addition, different A/M populations need to be screened to better ascertain the percentage of A/M cases due to mutations in *STRA6*. Continued recruitment of A/M patients is necessary for future genetic screenings.

## Appendix 1: Intronic and untranslated region sequence variants found in *STRA6*.

All non-coding sequence variants observed for *STRA6* are reported in this table. The novel 5 base pair insertion found 5′upstream of one of the alternate exons was found in 5/89 controls. ‘- -‘ denotes lack of sequence data for that corresponding sample. To access the data, click or select the words “Appendix 1.” This will initiate the download of a compressed (pdf) archive that contains the file.

## Appendix 2. Intronic and untranslated region sequence variants found in *SKI*.

All non-coding sequence variants observed for *SKI* are reported in this table. ‘- -‘ denotes lack of sequence data for that corresponding sample. To access the data, click or select the words “Appendix 2.” This will initiate the download of a compressed (pdf) archive that contains the file.
